# Involvement of c-Fos in cell proliferation, migration, and invasion in osteosarcoma cells accompanied by altered expression of Wnt2 and Fzd9

**DOI:** 10.1371/journal.pone.0180558

**Published:** 2017-06-30

**Authors:** Qiaozhen Wang, Huancai Liu, Qing Wang, Fenghua Zhou, Yongxin Liu, Yawen Zhang, Haoyu Ding, Meng Yuan, Fengjie Li, Yanchun Chen

**Affiliations:** 1 Department of Human Anatomy, Weifang Medical University, Weifang, Shandong, China; 2 Affiliated hospital, Weifang Medical University, Weifang, Shandong, China; 3 Department of Pathology, Weifang Medical University, Weifang, Shandong, China; 4 Department of Histology and Embryology, Weifang Medical University, Weifang, Shandong, China; University of South Alabama Mitchell Cancer Institute, UNITED STATES

## Abstract

Osteosarcoma (OS) is an aggressive bone tumor, and proto-oncogene c-Fos is involved in this lethal disease. However, the role and molecular mechanism of c-Fos in the development and progression of OS remain enigmatic. As one of the Wnt family members, Wnt2 is closely associated with the development of several malignant tumors. In the present study, the expression of c-Fos, Wnt2, and its receptor Fzd9 in human OS tissues, MG63 OS cell line, and human osteoblast hFOB 1.19 cell line was detected by Western blot analysis, immunohistochemical staining, or reverse transcription-polymerase chain reaction. The role of c-Fos in the OS was clarified by treating MG63 cells with small interfering RNA to knockdown c-Fos. Then, cell migration and invasion were assayed by transwell assays and wound healing assay; cell proliferation was assayed by MTS method and 5-ethynyl-2'-deoxyuridine DNA proliferation in vitro detection; cell apoptosis was assayed by flow cytometric method. Co-immunoprecipitation kit was used to confirm the relationship between c-Fos and Wnt2/Fzd9. We found that the expression of c-Fos, Wnt2, and Fzd9 protein was distinctly higher in human OS tissues than that in the adjacent non-cancerous tissues, and their expression in the MG63 OS cell line was markedly increased compared with that in the human osteoblast hFOB 1.19 cell line. Knockdown of c-Fos inhibited the proliferation, migration, and invasion of MG63 cells, and promoted the apoptosis of MG63 cells. Moreover, knockdown of c-Fos inhibited the expression of Wnt2 and Fzd9 mRNA and protein. Our data enforced the evidence that knockdown of c-Fos inhibited cell proliferation, migration, and invasion, and promoted the apoptosis of OS cells accompanied by altered expression of Wnt2 and Fzd9. These findings offer new clues for OS development and progression, and c-Fos may be a potential therapeutic target for OS.

## Introduction

Osteosarcoma (OS) is characterized by formation of cancerous bone tissue, early lung-targeted metastasis, and poor prognosis; OS is also an aggressive malignant tumor of the bone occurring in children and adolescents[1, 2]. The treatment of OS is largely dependent on surgery and chemotherapy; however, the therapeutic efficacy varies among patients because OS is prone to aggressive biological behavior and development of distant metastasis[3, 4]. In recent years, although considerable research on the mechanism of OS development and metastasis has been conducted, the molecular mechanisms are still unclear.

c-Fos is described as an immediate early response gene, and its encoded protein is an important transcription factor in eukaryotic cell[5]. c-Fos can induce the transcription and protein expression of downstream gene and regulate cell proliferation, apoptosis, migration, and invasion in malignant gliomar and breast cancer[6, 7]. Overexpression of c-Fos can interfere the dynamic equilibrium of cell proliferation and induce cell transformation and tumor formation[8]. As an activator protein-1 transcription factor, c-Fos can promote the occurrences of OS[9]. However, the role and molecular mechanism of c-Fos in the development and progression of OS remain uncertain.

Wnts comprise a large family of secreted signaling proteins that control cell proliferation, migration, and differentiation. They play a critical role in postnatal health and diseases, including cancers and degenerative diseases[10, 11]. Wnt2 is overexpressed and can promote tumorigenesis in many types of cancer[12]. As the receptor of Wnt2, Frizzled-9(Fzd9) is activated by Wnt2 and functions in Wnt signaling pathway[13]. In this study, we investigated the potential role and molecular mechanism of c-Fos in the development and progression of OS and the relationship among c-Fos, Wnt2, and Fzd9.

## Materials and methods

### Clinical samples and data

A total of 54 formalin-fixed and paraffin-embedded OS tissues and matched paracarcinoma tissues from the same specimens (from January 01, 2007 to December 30, 2014) were collected from the Affiliated Hospital of Weifang Medical University. All the specimens were collected with written informed consent prior to enrollment. The use of human tissues was approved by the Medical Ethics Committee of Weifang Medical University. No patients had received radiotherapy and chemotherapy prior to surgery.

### Cell culture

The MG63 human OS cell line was purchased from the cell bank of Shanghai Institute of Cell Biology (Shanghai, China) and cultured in modified Eagle’s medium (MEM, Gibco) supplemented with 10% fetal bovine serum (FBS, Gibco) at 37°C in a humidified atmosphere containing 5% CO_2_.

Human normal osteoblastic cell line hFOB 1.19 was purchased from the cell bank of Shanghai Institute of Cell Biology (Shanghai, China) and maintained in D-MEM/F-12 (Gibco) supplemented with 10% FBS (Gibco) and 0.3 mg/mL Geneticin (G418; Gibco) at 34°C in a humidified atmosphere containing 5% CO_2_.

### Immunohistochemical staining

After routine deparaffinization and hydration, tissue sections were treated with 0.01 M citrate buffer (pH 6.0) for antigen retrieval. Following 3% hydrogen peroxide incubation and 10% bovine serum albumin deprivation, slides were incubated with primary antibodies of c-Fos (1:100, Cell Signaling), Wnt2 (1:100, Abcam), and Fzd9 (1:100, Abcam) at 4°C overnight. Primary antibodies were recognized by the biotinylated secondary antibody and visualized by Vectastain avidin–biotin complex peroxidase system (Zsbio, China) and 3,3′-diaminobenzidine kit (DAB, Zsbio, China). The degrees of immunoreactivity were evaluated in accordance with our previous report[14]. We performed staining by only adding phosphate-buffered saline (PBS) instead of using primary antibodies to the sections as negative controls. The sections were photographed using an optical microscope (Olympus, BX53F, Japan) and then analyzed using the Image-Pro Plus 6.0 analytic system.

### Small interfering RNA transfection

Transfections of small interfering RNA (siRNA) and negative control (Ribobio, Guangzhou, China) were performed using Invitrogen Lipofectamine 3000 (Thermo Fisher Scientific) in accordance with the protocols of the manufacturer. The target sequence of c-Fos was 5′-GCA AGG TGG AAC AGT TAT C-3′. The specifications of c-Fos siRNA were as follows: sense was 5′-GCAAGGUGGAACAGUUAUC dTdT -3′ and anti-sense was 3′-dTdT CGU UCC ACC UUG UCA AUA G-5′. siRNA c-Fos and negative control were used at a final concentration of 60 nM. Other detailed procedures of transfection were as previously described[10].

### RNA isolation and reverse transcription-polymerase chain reaction

Total RNA was extracted from OS cell line and human normal osteoblastic cell line hFOB 1.19 using the Trizol kit (Ambion, USA) and 2 μg RNA was used for synthesis of complementary (c) DNA by reverse transcriptase (Promega, USA). The resulting complementary (c) DNA was used for PCR analysis using EsTaq MasterMix (CWBIO, China). The following primers (Sangon Biotech, China) were used: forward 5′-TAC TAC CAC TCA CCC GCA GAC T-3′ and reverse 5′-GAA TGA AGT TGG CAC TGG AGA C-3′ for c-Fos; forward 5′-GAC CAA GTG TGG GTG TAA GTT C-3′ and reverse 5′-ATG TAG CGG TTG TCCAGT CAG-3′ for Wnt2; forward 5′-CGG CAC CAA CAC AGA GAA G-3′ and reverse, 5′-CGT AGA CAT AGC AAA CGA TGA C-3′ for Fzd9; and forward 5′-TGA CGT GGA CAT CCG CAA AG-3′ and reverse 5′-CTG GAA GGT GGA CAG CGA GG-3′ for β-actin. PCR was performed under the following conditions: 94°C for 3 min, followed by 30 cycles of 94°C for 30 s, 57°C for 30 s, and 72°C for 30 s, and finally 72°C for 5 min.

### Western blot analysis

Total protein was extracted from OS cell lines and human normal osteoblastic cell line hFOB 1.19 using the RIPA buffer containing protease inhibitor cocktail (Sigma, USA), and 90 μg of total protein per well was used for Western blot in accordance with the protocols of the manufacturer. The primary antibodies of c-Fos (1:1000, Cell Signaling), Wnt2 (1:1000, Abcam), and Fzd9 (1:1000, Abcam) incubated at 4°C for overnight and the appropriate horseradish peroxidase-linked secondary antibody (1:20000, Jackson Immuno-Research, USA) were used to visualize the immunoreactivity. The house-keeping protein GAPDH was used as an internal control. The intensity of each band was measured with the Image-Pro Plus 6.0 analytic system.

### Immunofluorescence

MG63 cells were treated with siRNA c-Fos or negative control after being planted on coverslips for 18 h. At 24 and 48 h after transfection, the immunofluorescences of MG63 cells were performed according to our previous protocol[15]. The primary antibodies used were as follows: rabbit anti-c-Fos IgG (1:100, Cell Signaling), rabbit anti-Wnt2 IgG (1:100, Abcam), and rabbit anti-Fzd9 IgG (1:100, Abcam). Subsequently, the nuclei were counterstained with Hoechst33258 (1:1000; Sigma-Aldrich, USA). Images were obtained using a fluorescence microscope (Olympus, BX53F, Japan). PBS was used as negative control instead of primary antibodies.

### MTS assay

Cell viability was detected by MTS assay (Promega, USA). At 18 h after transfection, cells were plated into a 96-well plate at a density of 5 × 10^3^ to 1 × 10^4^ cells/well. Incubation was continued for detecting the cell viability at 24, 48, 72, 96, and 120 h after transfection. MTS (20 μL) was diluted with 100 μL medium, and then the mixture was pipetted into each well and incubated for 1 h at 37°C with 5% CO_2_. The absorbance was recorded immediately at 490 nm using a microplate reader (Thermo Scientific, Multiskan FC, USA).

### Flow cytometric

Flow cytometry analysis of apoptosis Annexin V-FITC was used in conjunction with a vital dye PI to distinguish apoptotic cells (Annexin V-FITC-positive, PI-negative) from necrotic cells (Annexin V-FITC-positive, PI-positive). Annexin V-FITC bind phosphatidylserine residues translocated from the inner to the outer leaflet of the plasma membrane during the early stages of apoptosis. In brief, MG63 cells were plated in six-well plate at the density of 20 × 10^4^ cells/well and were treated with siRNA c-Fos or negative control after being planted for 18 h. At 24, 48, and 72 h after transfection, the cells were detached by incubation with tripsin (without EDTA), washed in PBS, and collected by centrifugation. Then, the cells were incubated with 100 μL Annexin V-FITC and 100 μL PI for 30 min at room temperature, washed, and resuspended in the binding buffer. Viable cells, early apoptotic cells, late apoptotic cells, and necrotic cells were detected by flow cytometer (BD Bioscences). For each sample, 2 × 10^4^ cells were acquired. Analysis was carried out by triplicate determination on three separate experiments.

### EdU DNA proliferation in vitro detection

MG63 Cells were treated with siRNA c-Fos or negative control after being planted for 18 h. Following the manual of a 5-ethynyl-2'-deoxyuridine (EdU) labeling/detection kit (Ribobio, China), 50 μM EdU labeling medium was added to the cells for 2 h at 37°C in a humidified atmosphere with 5% CO_2_ at 24 and 48 h after transfection. Thereafter, cultured cells were fixed with 4% paraformaldehyde (pH 7.4) for 15 min and incubated with glycine for 5 min. After being washed with PBS, cultured cells were stained with anti-EdU working solution at room temperature for 30 min. Following washing with 0.5% TritonX-100 in PBS, the cells were incubated with 5 μg/mL Hoechst 33342 dye at room temperature for 20 min. Images were obtained using an inverted fluorescence microscope (Leica Microsystems CMS GmbH). The percentage of EdU-positive cells was calculated from five random fields, and all experiments were repeated three times.

### Transwell migration and invasion assays

For migration assay, 5 × 10^4^ transfected cells were placed in the upper chamber of each insert. For invasion assay, 5 × 10^5^ transfected cells were placed on the upper chamber of each insert pre-coated with Matrigel. The lower chamber of the transwell was then filled with MEM with 20% FBS. After incubation for migration assays and invasion assays, the upper surface of the membrane was wiped with a cotton tip and cells attached to the lower surface were stained with crystal violet. The images of invaded or migrated cells were photographed and the number was counted in five random fields. Data of the average number of cells were from three independent experiments.

### Wound healing assay

A wound healing assay was used to determine cell migration. Transfected cells were cultured to full confluence and a wound of 1 mm width was made with a plastic scriber. Cells were then washed with PBS and cultured at 37°C with 5% CO2 for another 48 h. Finally, cells in each group were observed under a microscope (Leica Microsystems CMS GmbH) at 0, 24, and 48 h after transfection. The distance of cell migration was measured in five random fields. All samples were tested in triplicate.

### Co-immunoprecipitation kit

Co-immunoprecipitation (Co-IP) was performed using Co-IP Kit (Thermo Scientific, USA) in the presence of protease inhibitor cocktail (Sigma, USA), following the protocols of the manufacturer. Part of the lysate was saved as the control “input”. The remaining lysate was sequentially incubated with c-Fos antibody (Cell Signaling) in accordance with the protocols of the manufacturer. The protein was interacted with c-Fos/protein A agarose beads and was saved as “Co-IP”, and the supernatant was saved as “flow.” The three groups of protein were assessed by Western blot analysis with Wnt2 (Abcam) and Fzd9 (Abcam) antibodies to assess enrichment in Wnt2 and Fzd9 bound proteins.

### Statistical analysis

All statistical analyses were conducted using the SPSS 16.0 software. The data were presented as mean ± standard deviation (SD). Two groups were compared using a two-tailed paired Student’s t-test. The Chi-square test was used to analyze the relationship among c-Fos, Wnt2, and Fzd9 expression and clinicopathologic characteristics. In all cases, *p* < 0.05 was considered statistically significant.

## Results

### Expression of c-Fos, Wnt2, and Fzd9 protein was distinctly higher in human OS tissues than in the adjacent non-cancerous tissues

**Fig 1:**
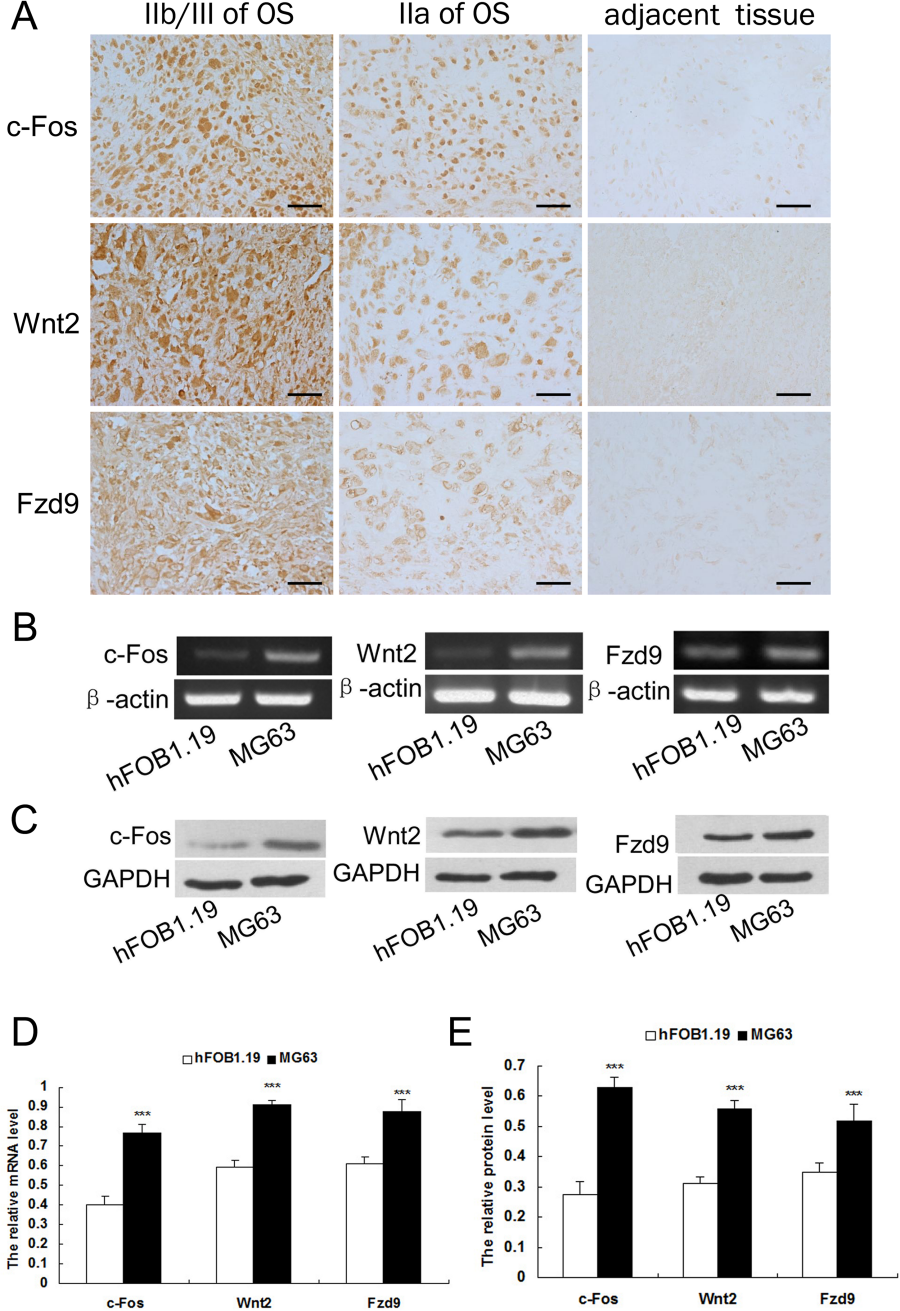
High expression of c-Fos, Wnt2, and Fzd9 in human OS tissues and MG63 OS cell line. A. Expression of c-Fos, Wnt2, and Fzd9 protein in human OS tissues were distinctly higher than that in the adjacent non-cancerous tissues detected by IHC staining, bar = 50 μm. B. The mRNA levels of c-Fos, Wnt2, and Fzd9 in the OS MG63 cell line were markedly up-regulated compared with those in hFOB 1.19 cell line examined by RT-PCR. β-actin served as an internal control. C. The protein levels of c-Fos, Wnt2, and Fzd9 in the OS MG63 cell line were markedly up-regulated compared with those in hFOB 1.19 cell line examined by Western blot. GAPDH served as an internal control. D. Data represented means ± SD for four independent experiments of RT-PCR analysis of c-Fos, Wnt2, and Fzd9. E. Data represented means ± SD for four independent experiments of Western blot analysis of c-Fos, Wnt2, and Fzd9. ***p < 0.001 versus hFOB1.19 group.

Expression of c-Fos, Wnt2, and Fzd9 was examined by immunohistochemical (IHC) staining. The results showed that the expression of c-Fos was higher in OS tissues than in the adjacent non-cancerous tissues (Fig 1A). We then analyzed the associations of c-Fos expression with clinicopathological parameters of OS tissues. As shown in Table 1, the rates of high c-Fos expression in stages IIa and IIb/III of OS tissues were 32.0% and 75.9%, respectively (*p* < 0.01). These findings suggested that the level of c-Fos expression was correlated with tumor stages.

**Table 1:** Associations of the expression of c-Fos, Wnt2, and Fzd9 with tumor clinical stages.

	IIa stage (n = 25)	IIb/III stage (n = 29)	*p*-value
c-Fos expression			
High(n, %)	8(32.0)	22(75.9)	*p*<0.01**
Low(n, %)	17(68.0)	7(24.1)	
Wnt2 expression			
High(n, %)	15(60.0)	27(93.1)	*p*<0.01**
Low(n, %)	10(40.0)	2(6.8)	
Fzd9 expression			
High(n, %)	7(28.0)	20(68.9)	*p*<0.01**
Low(n, %)	18(72.0)	9(31.1)	

**With significant difference

Our results also showed that the expression of Wnt2 and Fzd9 was higher in OS tissues than in the adjacent non-cancerous tissues (Fig 1A); this trend was in agreement with that found in the expression of c-Fos. The rates of high-level Wnt2 expression in stages IIa and IIb/III of OS tissues were 60.0% and 93.1%, respectively (*p* < 0.01) (Table 1). The rates of high-level Fzd9 expression in stages IIa and IIb/III OS were 28.0% and 68.9%, respectively (*p* < 0.01) (Table 1). The high level expression of Wnt2 and Fzd9 occurred more frequently in later stage than in the early stage of OS. The expression of Wnt2 and Fzd9 was also correlated with the tumor stages.

### Expression of c-Fos, Wnt2, and Fzd9 in the OS MG63 cell line was markedly increased compared with that in the human osteoblast hFOB 1.19 cell line

In confirming whether the expression of c-Fos, Wnt2, and Fzd9 was associated with OS, reverse transcription-polymerase chain reaction (RT-PCR) and Western blot techniques were used to detect their expression in the OS MG63 cell line and human osteoblast hFOB 1.19 cell line. Our data showed that the mRNA and protein levels of c-Fos, Wnt2, and Fzd9 in the OS MG63 cell line were markedly up-regulated compared with those in hFOB 1.19 cell line (*p* < 0.001) (Fig 1B–1D). Furthermore, the expression of c-Fos, Wnt2, and Fzd9 was increased in the OS.

### Knockdown of c-Fos in MG63 cells in vitro

**Fig 2:**
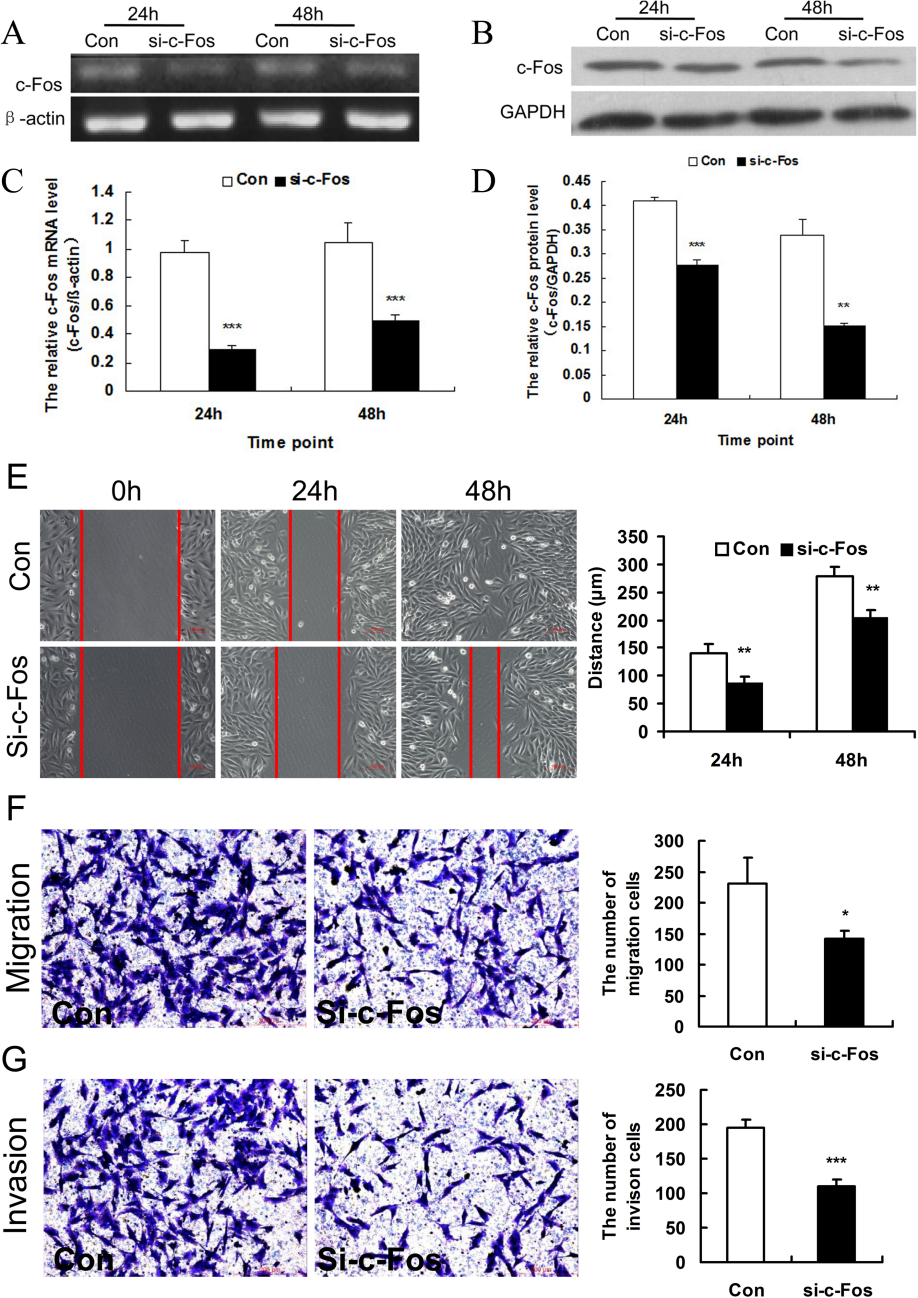
Knockdown of c-Fos inhibited cell migration and invasion of MG63 cells in vitro. A. Expression of c-Fos in MG63 cells was examined by RT-PCR at 24 and 48 h after transfection with c-Fos siRNA (si-c-Fos) or the negative control (Con). β-actin served as an internal control. B. Expression of c-Fos was examined by Western blot at 24 and 48 h after transfection with c-Fos siRNA (si-c-Fos) or the negative control (Con). GAPDH served as an internal control. C. Data represented means ± SD for four independent experiments of RT-PCR analysis of c-Fos. D. Data represented means ± SD for four independent experiments of Western blot analysis of c-Fos. E. Wound healing assay for determining the migration of MG63 cells transfected with c-Fos siRNA (si-c-Fos) or negative control (Con), bar = 100 μm; Data of the distance of cell migration were from three independent experiments. F. Images showing that the migrated cells treated with c-Fos siRNA (si-c-Fos) were significantly decreased compared with the control groups, bar = 200 μm. Data of the average number of cells was from three independent experiments. G. Images showing that the invasive cells treated with c-Fos siRNA (si-c-Fos) were significantly decreased compared with the control groups, bar = 200 μm. Data of the average number of cells were from three independent experiments. *p < 0.05, **p < 0.01, ***p < 0.001 versus negative control (Con).

MG63 cells were transfected with c-Fos siRNA/negative control and then the expression of c-Fos was detected by RT-PCR and Western blot. The expression levels of c-Fos mRNA and protein in MG63 cells treated with c-Fos siRNA were obviously lower than those treated with negative control at 24 and 48 h (*p* < 0.001, *p* < 0. 01) after transfection. c-Fos siRNA effectively inhibited the expression of c-Fos in OS cells (Fig 2A–2D).

### Knockdown of c-Fos inhibited the migration and invasion of MG63 cells in vitro

In investigating the role of c-Fos on the migration and invasion of OS cells, the transwell assay and wound healing assay were used to determine cell migration and invasion at 0, 24, or 48 h after transfection. The result of wound healing assay showed that the migration distances of cells treated with negative control were 141.0 ± 12.9 and 278.7 ± 14.0 μm at 24 and 48 h after transfection, and those of the cells treated with c-Fos siRNA were 87.4 ± 8.4 (*p* < 0.01) and 206.2 ± 9.2 μm (*p* < 0.01) (Fig 2E).

The result of the transwell migration assay showed that the number of cells treated with negative control was 231.7 ± 40.4, while the number of cells treated with c-Fos siRNA was 141.1 ± 13.7 (*P* < 0.05) (Fig 2F). The result of the transwell invasion assay showed that the number of cells treated with negative control was 195.2 ± 11.3, while the number of cells treated with c-Fos siRNA was 109.4 ± 9.8 (*p* < 0.001) (Fig 2G).

All these findings suggested that knockdown of c-Fos inhibited the migration and invasion of MG63 cell in vitro.

### Knockdown of c-Fos inhibited the proliferation and promoted the apoptosis of MG63 cells in vitro

**Fig 3:**
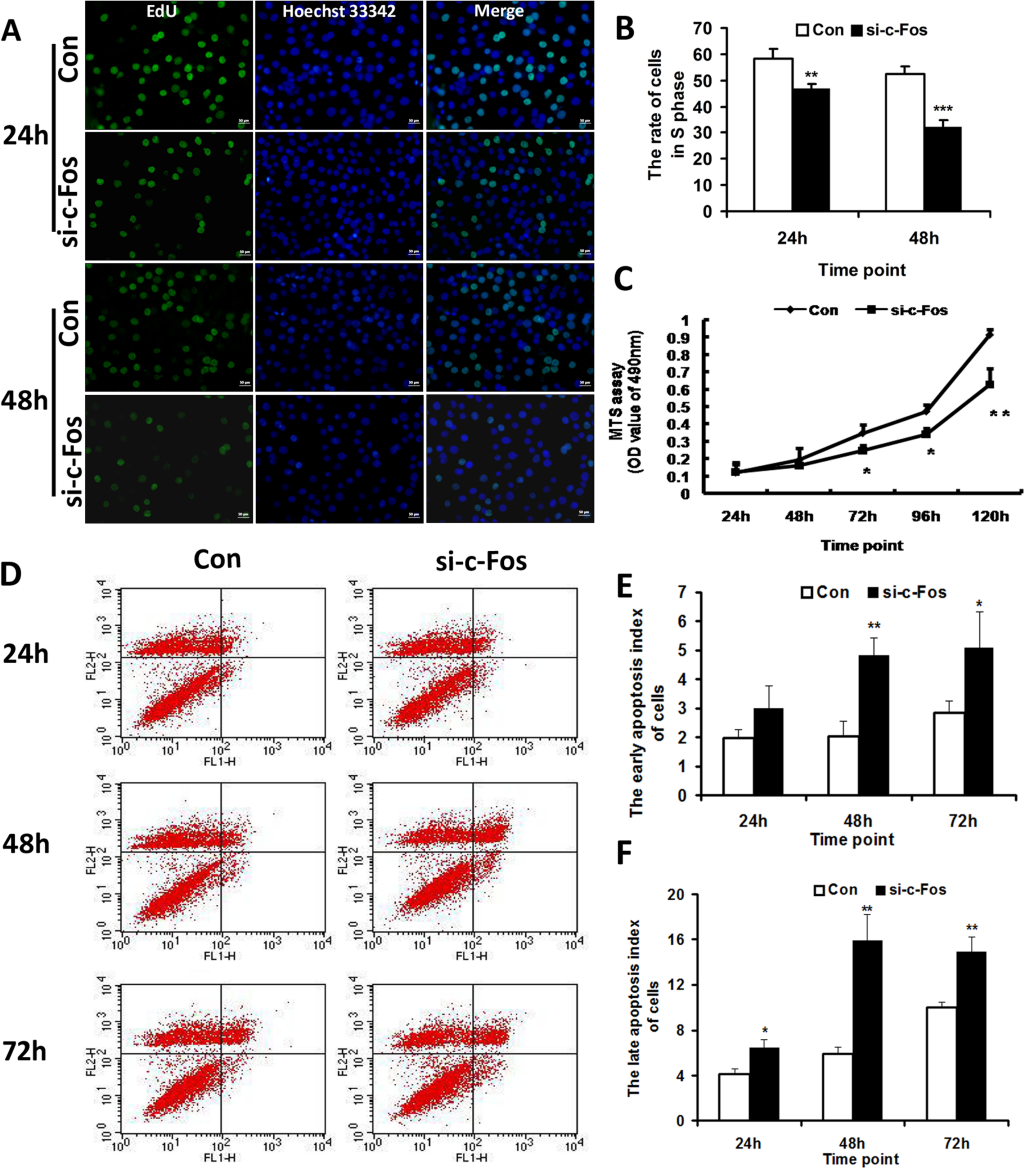
Knockdown of c-Fos inhibited the proliferation and promoted the apoptosis of MG63 cells. A. Images of EdU DNA proliferation in vitro detection showing the cells in S phase treated with c-Fos siRNA (si-c-Fos) or the negative control (Con). bar = 50 μm. B. The rate of cells in S phase. Data represented means ± SD for three independent experiments of EdU DNA proliferation in vitro detection. C. MTS assay demonstrated that silencing of c-Fos inhibited the cell proliferation capability of MG63 cells on the indicated time points after transfection with c-Fos siRNA (si-c-Fos). D. Flow cytometer analysis showing that the apoptosis index of cells treated with c-Fos siRNA (si-c-Fos) was significantly increased compared with the control group. E. The early apoptosis index of cells. Data were from three independent experiments of flow cytometer analysis. F. The late apoptosis index of cells. Data were from three independent experiments of flow cytometer analysis. *p < 0.05, **p < 0.01, ***p < 0.001 versus negative control (Con).

To investigate the effect of c-Fos on the proliferation of OS cells, EdU DNA proliferation in vitro detection was performed. At 24 and 48 h after transfection, we used an inverted fluorescence microscope to obtain the rate of cells in different phases. The proportion of cells treated with negative control in the S phase was 58.4% ± 3.8%, and the proportion of cells treated with c-Fos siRNA in the S phase was 47.0% ± 1.7% at 24 h (*p* < 0.01), while the proportion of cells treated with negative control in the S phase was 52.3% ± 3.1% and the proportion of cells treated with c-Fos siRNA in the S phase was 32.3% ± 2.4% at 48 h (*p* < 0.001) (Fig 3A and 3B). These results indicated that cells treated with c-Fos siRNA had lower proliferation rate than the control. Knockdown of c-Fos inhibited the proliferation of MG63 cells in vitro.

To further confirm the effect of c-Fos on the proliferation of OS cells, the MTS assay was performed at 24, 48, 72, 96, and 120 h after transfection. The results showed that absorbance value at 490 nm of MG63 cells transfected with c-Fos siRNA was lower than that of MG63 cells transfected with the negative control at 72 (*p* < 0.05), 96 (*p* < 0.05), and 120 h (*p* < 0.01) after transfection (Fig 3C). Therefore, knockdown of c-Fos inhibited MG63 cell proliferation.

To confirm the apoptosis occurrence upon treatment of c-Fos siRNA, we assessed apoptosis by flow cytometer analysis after double labeling with Annexin V and PI. Fig 3 showed that at 24, 48, and 72 h after transfection, the early apoptosis indexes of cells treated with negative control were 1.9 ± 0.3, 2.1 ± 0.5, 2.8 ± 0.4 respectively, while those for the cells treated with c-Fos siRNA were 3.0 ± 0.8 (*p* > 0.05), 4.8 ± 0.6 (*p* < 0.01), and 5.1 ± 1.2 (*p* < 0.05) (Fig 3D and 3E). The late apoptosis indexes of cells treated with negative control were 4.2 ± 0.5, 5.9 ± 0.6, 10.0 ± 0.5 and those for the cells treated with c-Fos siRNA were 6.4 ± 0.8 (*p* < 0.05), 16.0 ± 2.3 (*p* < 0.01), and 14.9 ± 1.4 (*p* < 0.01) at 24, 48, and 72 h after transfection (Fig 3D and 3F). These results indicated that cells treated with c-Fos siRNA had higher apoptosis rate than the control and that knockdown of c-Fos promoted the apoptosis of MG63 cells in vitro.

### Knockdown of c-Fos down-regulated the expression of Wnt2 and its receptor Fzd9 in MG63 cells in vitro

**Fig 4:**
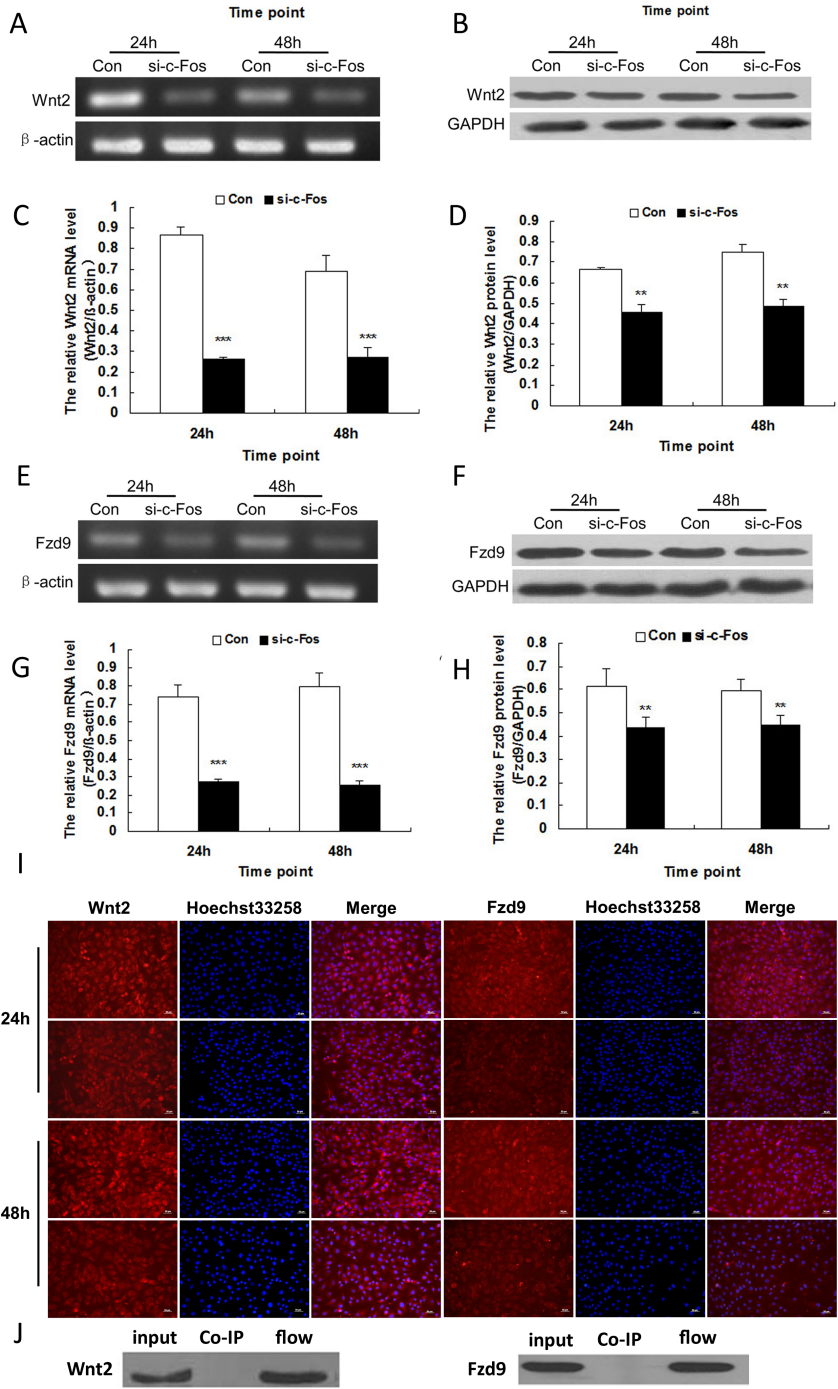
Knockdown of c-Fos down-regulated the expression of Wnt2 and its receptor Fzd9 in MG63 cells at 24 and 48 h after transfection with c-Fos siRNA (si-c-Fos) and the negative control (Con). A. Expression of Wnt2 was examined by RT-PCR. β-actin served as an internal control. B. Expression of Wnt2 was examined by Western blot. GAPDH served as an internal control. C. Data represented means ± SD for four independent experiments of RT-PCR analysis of Wnt2. D. Data represented means ± SD for four independent experiments of Western blot analysis of Wnt2. E. Expression of Fzd9 was examined by RT-PCR. β-actin served as an internal control. F. Expression of Fzd9 was examined by Western blot. GAPDH served as an internal control. G. Data represented means ± SD for four independent experiments of RT-PCR analysis of Fzd9. H. Data represented means ± SD for four independent experiments of Western blot analysis of Fzd9. I. The expression of Wnt2 and its receptor Fzd9 detected by immunofluorescence staining, bar = 50 μm. J. Wnt2 and Fzd9 were enriched in “flow” group, but no bands were found in Co-IP group. No direct connection existed between c-Fos and Wnt2/Fzd9. **p < 0.01, ***p < 0.001 versus negative control (Con).

To examine the potential role of c-Fos in regulating Wnt signaling pathway, we detected the mRNA and protein expression of Wnt2 and its receptor Fzd9 by RT-PCR and Western blot after knockdown of c-Fos in MG63 cells. The mRNA and protein levels of Wnt2 and Fzd9 in the MG63 cells treated with c-Fos siRNA were markedly reduced compared with those for the cells treated with negative control siRNA at 24 and 48 h after transfection (*p* < 0.01) (Fig 4A–4H). The immunofluorescence results also showed that the immunoreactivity of Wnt2 and Fzd9 decreased obviously after knockdown of c-Fos in MG63 cells (Fig 4I); this trend was in agreement with that observed in the results of Western blot.

### c-Fos did not combine with Wnt2 and Fzd9 directly in vitro

In investigating the relationship between the c-Fos and Wnt2/Fzd9, we used the Co-IP Kit to detect the expression of Wnt2 and Fzd9 after immunoprecipitation with antibody against c-Fos (Fig 4J). Immunoprecipitation assays showed that the expression of Wnt2 and Fzd9 of “Co-IP” group of MG63 cell lysate was negative and the expression of “flow” group was positive after incubation of MG63 cell lysate with anti-c-Fos antibody. Therefore, no direct connection existed between c-Fos and Wnt2/Fzd9.

## Discussion

The prognosis of OS is poor because of the early lung-targeted metastasis. As an oncogene, c-Fos is associated with the proliferation, apoptosis, migration, and invasion of tumor cells in various human cancers, including hepatocellular carcinoma, breast cancer, and prostate cancer[16, 17]. c-Fos play an oncogenic role through cooperating with c-jun in OS, and overexpression of c-Fos increased the risk of chromatin reorganization in human OS cells[18]. However, the role of c-Fos in the development and progression of OS remains unclear.

The present study was conducted to explore the correlation between c-Fos and the occurrence and development of OS and its potential mechanism. The research data revealed that the expression of c-Fos was distinctly increased in OS tissues and MG63 cells, and the high expression of c-Fos was correlated with tumor stages. In other words, c-Fos was closely related to the pathogenesis of OS. In the study of mechanism in vitro, knockdown of c-Fos inhibited the proliferation, migration, and invasion, as well as induced apoptosis of MG63 cells. The imbalance of cell proliferation and apoptosis might be an important mechanism of tumorigenesis. Migration and invasion abilities are important for the metastasis of tumor cells. The change of cell migration and invasion might be an important cause of OS progression, and c-Fos was a key regulator in the occurrence and development of OS.

We also found that the expression of Wnt2 and Fzd9 in human OS tissues was in accordance with c-Fos and that the knockdown of c-Fos reduced the expression of Wnt2 and Fzd9 in vitro. All the results suggested that c-Fos might play an important role in regulating the expression of Wnt2 and Fzd9 in the development and progression of OS. Previous pieces of evidence revealed that Wnt signaling pathway was involved in the proliferation, migration, invasion, and apoptosis of cancers, such as lung cancer[19]. Moreover, several Wnt proteins play a role in regulating bone masses, including Wnt1, Wnt3a, Wnt4, Wnt5, Wnt5a, Wnt7a, Wnt10b, and Wnt14[20, 21]. In addition, Haydon et al.[22] demonstrated that the Wnt pathway was linked to OS, and the staining of human OS tumor cells showed the accumulation of β-catenin and activation of Wnt pathway. The proteins of Wnt4, Wnt 5a, Wnt7a, and Wnt14 have been studied in the OS cell lines, implying that Wnt pathway may serve as a tumor activator[23]. Enhanced Wnt signaling by methylation-mediated loss of SFRP2 promoted osteosarcoma cell invasion[24]. We also reported previously that Wnt1 and its receptor Fzd1 were upregulated in human OS tissues and MG63 OS cells[14]. Moreover, Wnt/β-catenin signaling pathway is associated with the erosion and migration of OS SAOS-2 cells[25]. Wnt2 is implicated in various human cancers and upregulated in human cancer and contributed to tumorigenesis[26]. Wnt2 promotes gastrointestinal tract cancer and non-small cell lung cancer progression by activating Wnt/β-catenin pathway[27]. Previous studies also found that the ectopic expression of Wnt2 plays an important role in the proliferation, invasion, and migration of breast cancer[28]. However, Wnt2 and its receptor Fzd9 in OS have not been explored yet. Our data showed that c-Fos regulated cell biological function in the proliferation, migration, invasion, and apoptosis of MG63 cells, accompanied by altered expression of Wnt2 and Fzd9.

The development of OS is a complex process because of numerous factors[29], such as the imbalance of suppressor genes and oncogenes[30]. Clinical observations over the past few decades indicated that any single gene therapy method cannot fully solve OS. Our study was conducted to explore the relationship of c-Fos and the genes of Wnt signaling pathway. However, c-Fos did not combine with Wnt2 and Fzd9 directly. Another complex relationship might exist between them. Further studies are required to fully elucidate the mechanism of c-Fos in OS, and to verify the mechanism of the knockdown of c-Fos in decreasing the expression of Wnt2 and Fzd9.

In this report, we presented powerful evidences that high expression of c-Fos, Wnt2 and Fzd9 was found in human OS tissues and MG63 cells, and the level of c-Fos, Wnt2 and Fzd9 expression was correlated with tumor stages. Knockdown of c-Fos inhibited the proliferation, migration, and invasion, as well as promoted the apoptosis of MG63 cells. These results highlighted that c-Fos played a critical role in the development and progression of OS. In addition, we found that during the genesis and development of OS, the expression change of c-Fos was accompanied by altered expression of Wnt2 and Fzd9 in MG63 cells. There are somehow unknown correlations between c-Fos expression and Wnt2/Fzd9 expression. In the future, we will further explore the relationship between c-Fos and Wnt signaling pathways and offer more clues for clarifying OS development and progression. c-Fos may be a potential therapeutic target for OS.

## Supporting information

S1 Fig Data on the relative mRNA and protein levels of c-Fos, Wnt2 and Fzd9 in hFOB1.19 cell line and MG63 OS cell line. D. Data on the relative mRNA level of c-Fos, Wnt2 and Fzd9 analyzed by RT-PCR (n = 4). E. Data on the relative protein level of c-Fos, Wnt2 and Fzd9 analyzed by Western blot (n = 4).
(XLSX)

S2 Fig Data on the migration and invasion of MG63 cells after transfected with c-Fos siRNA (si-c-Fos) or the negative control (Con). C. Data on the relative mRNA level of c-Fos analyzed by RT-PCR (n = 4). D. Data on the relative protein level of c-Fos analyzed by Western blot (n = 4). E. Data of wound healing assay on migration distance (n = 3), and five visions were taken by random in each experiment. F. Data of transwell assay on the average number of migration cells (n = 3), and five visions were taken by random in each experiment. G. Data of transwell assay on the average number of invasion cells (n = 3), and five visions were taken by random in each experiment.
(XLSX)

S3 Fig Data on the proliferation and apoptosis of MG63 cells after transfected with c-Fos siRNA (si-c-Fos) or the negative control (Con). B. Data of EdU assay on the rate of cells in S phase (n = 3), and five visions were taken by random in each experiment. C. Data of MTS assay on the absorbance of cells at 490 nm (n = 3), and five repetitions were taken in each experiment. E. Data of flow cytometric analysis on the early apoptosis index of cells (n = 3). F. Data of flow cytometric analysis on the late apoptosis index of cells (n = 3).
(XLSX)

S4 Fig Data on the relative mRNA and protein levels of Wnt2 and Fzd9 in MG63 cells after transfected with c-Fos siRNA (si-c-Fos) or the negative control (Con). C. Data on the relative mRNA level of Wnt2 analyzed by RT-PCR (n = 4). D. Data on the relative protein level of Wnt2 analyzed by Western blot (n = 4). G. Data on the relative mRNA level of Fzd9 analyzed by RT-PCR (n = 4). H. Data on the relative protein level of Fzd9 analyzed by Western blot (n = 4).
(XLSX)

S1 Table Associations of the expression of c-Fos, Wnt2, and Fzd9 with tumor clinical stages. Comparison of the expression of c-Fos, Wnt2, and Fzd9 in human OS tissues of stage IIa and stage IIb/III.
(XLS)
